# Comparison of restrictive fluid therapy with goal-directed fluid therapy for postoperative delirium in patients undergoing spine surgery: a randomized controlled trial

**DOI:** 10.1186/s13741-021-00220-5

**Published:** 2021-12-15

**Authors:** Duo Duo Wang, Yun Li, Xian Wen Hu, Mu Chun Zhang, Xing Mei Xu, Jia Tang

**Affiliations:** 1 Department of Anesthesiology, The Second Hospital of Anhui Medical University, 678 Furong Road, Economic Development Zone, Hefei City, 230032 Anhui Province China; 2 Key Laboratory of Anesthesiology and Perioperative Medicine of Anhui Higher Education Institutes, Anhui Medical University, Hefei city, 230032 Anhui China

**Keywords:** Restrictive fluid therapy, Goal-directed fluid therapy, Spinal surgery, Postoperative delirium

## Abstract

**Background:**

Postoperative delirium (POD) is a common phenomenon after spinal surgery. Intraoperative fluid management may affect POD. The aim of this study was to compare the effects of restrictive fluid therapy (RF) with those of goal-directed fluid therapy (GDT) on POD.

**Methods:**

A total of 195 patients aged ≥ 50 years who underwent spinal surgery were randomly divided into two groups: the RF group and the GDT group. In group RF, a bolus of lactated Ringer’s solution was administered at a dose of 5 mL·kg^-1^ before the induction of anesthesia, followed by a dose of 5 mL·kg^-1^·h^-1^ until the end of surgery. For patients in the GDT group, in addition to the initial administration of lactated Ringer’s solution at 5 mL·kg^-1^, the subsequent fluid therapy was adjusted by using a continuous noninvasive arterial pressure (CNAP) monitoring system to maintain pulse pressure variation (PPV) ≤ 14%. The primary endpoint was the incidence of POD, assessed once daily with the Confusion Assessment Method-Chinese Reversion (CAM-CR) scale at 1–3 days postoperatively. The secondary endpoints were intraoperative fluid infusion volume, urine volume, mean arterial pressure (MAP), heart rate (HR), cardiac index (CI), regional cerebral oxygen saturation (rSO_2_) value, lactic acid value, and visual analog scale (VAS) pain score at 1–3 days after surgery. Moreover, postoperative complications and the length of hospital stay were recorded.

**Results:**

The incidence of POD was lower in the GDT group than in the RF group (12.4% vs 4.1%; *P* = 0.035) in the first 3 days after spine surgery. Compared to group RF, group GDT exhibited a significantly increased volume of intraoperative lactated Ringer’s solution [1500 (interquartile range: 1128 to 1775) mL vs 1000 (interquartile range: 765 to 1300) mL, *P* < 0.001] and urine volume [398 (interquartile range: 288 to 600) mL vs 300 (interquartile range: 200 to 530) mL, *P* = 0.012]. Intraoperative MAP, CI and rSO_2_ values were higher in the GDT group than in the RF group (*P* < 0.05). Moreover, the length of hospital stay [17.0 (14 to 20) days versus 14.5 (13 to 17.0) days, *P* = 0.001] was shorter in the GDT group than in the RF group.

**Conclusions:**

GDT reduced the incidence of POD in middle- and old-aged patients undergoing spinal surgery possibly by stabilizing perioperative hemodynamic and improving the supply and demand of oxygen.

**Trial registration:**

ChiCTR2000032603; Registered on May 3, 2020.

## Introduction

Postoperative delirium (POD), an acute condition characterized by reduced awareness of the environment and a disturbance in attention, typically occurs between 24 and 72 h after surgery (American Society of Psychiatrists.DSM-5 Criteria for Delirium 2013; Miller et al. 2018). A previous study suggested that the incidence of POD in patients undergoing spinal surgery is up to 40.5% for older adults (Brown et al. 2016) and approximately 9.3% for middle- and old-aged adults (Jiang et al. 2017). The consequences of POD can be profound, including an increased risk of prolonged hospitalization and need for extended care, resulting in functional decline, postoperative cognitive dysfunction, and increased mortality (Witlox et al. 2010; Aitken et al. 2017). The risk of POD is multifactorial. Some studies and consensus-based guidelines on POD have suggested that POD may be the result of pathological changes in the central nervous system induced and aggravated by operative trauma, anesthesia-related stress, and other external factors, which are related to changes in the tissue perfusion and brain oxygen supply during the perioperative period (Hughes et al. 2012; Aldecoa et al. 2017; Cascella et al. 2018).

Intraoperative fluid management is considered an important factor affecting the tissue perfusion and brain oxygen supply in surgical patients (Makaryus et al. 2018). The amount of intraoperative fluid management has been described as a modifiable risk factor of POD (Brown et al. 2016; Mailhot et al. 2019). Some studies have demonstrated that aggressive or “liberal” intraoperative fluid therapy is linked to increased postoperative complications and length of hospital stay in patients undergoing spine surgery, and additional evidence has suggested that more restrictive fluid protocol leads to fewer complications and a shorter hospital stay (Siemionow et al. 2012a, b; Hart et al. 2013). However, overly restricted or inadequate fluid therapy leads to hypotension, organ hypoperfusion with successive organ dysfunction and failure, and even adverse outcomes (Schol et al. 2016; Myles et al. 2019), which will increase the complication rate, hospital stay, and mortality.

Recently, goal-directed fluid therapy (GDT), defined as the deliberate and individualized optimization of hemodynamics and oxygen delivery using fluid and/or vasoactive infusions, has been proposed (Makaryus et al. 2018). GDT, guided by dynamic indicators of fluid responsiveness (e.g., pulse pressure variation, PPV), has been shown to decrease postoperative complications and to shorten the length of stay during major abdominal surgery (Coeckelenbergh et al. 2019). Compared with traditional fluid therapy, GDT can effectively maintain the stability of perioperative hemodynamics, optimize the relationship between tissue perfusion, and may reduce the incidence of POD in spinal surgery (Zhang et al. 2018).

However, few studies have examined the comparative outcome performances of restrictive and GDT in major spinal surgery. Therefore, we conducted a prospective, randomized, controlled trial to compare the incidence of POD in patients who were undergoing elective major spinal surgery with either intraoperative restrictive or GDT.

## Materials and methods

### Study design

This randomized clinical study was performed according to the Declaration of Helsinki principles between May 2020 and November 2020 in the Second Affiliated Hospital of Anhui Medical University. The study was approved by the Ethics Committee for Clinical Trials of the Second Affiliated Hospital of Anhui Medical University, Hefei, China [approval no.: PJ-YX2019-052(F2)]. The trial was registered in the Chinese Clinical Trial Registry before patient enrollment (ChiCTR2000032603). Written informed consent was obtained from each subject.

Inclusion criteria: (1) Age ≥ 50 years old, (2) lumbar spine and/or thoracic spinal stenosis surgery, (3) American Society of Anesthesiologists (ASA) grade of I–II, (4) body mass index (BMI) < 30 kg·m^-2^, (5) preoperative hematocrit > 0.30, (6) effective communication with the physician. Exclusion criteria: (1) patients with a history of mental illness or neurological disease; (2) patients receiving drugs that may affect cognitive function; (3) patients with visual, auditory, or language communication disorders; (4) patients with liver and kidney dysfunction and severe cardiopulmonary disease; and (5) patients who failed to pass the preoperative simple mini-mental state scale (MMSE) exam (patients with illiteracy who obtained a score ≤ 17, patients with 1–6 years of education who obtained a score ≤ 20, patients with greater than 7 years of education who obtained a score ≤ 23) (Li et al. 2016).

A statistician, who was independent of data management and statistical analyses, generated random numbers (in a 1:1 ratio) using the SPSS 21.0 (SPSS Inc., Chicago, IL, USA). The results of randomization were sealed in sequentially numbered envelopes and stored at the site of the investigation until interventions were assigned. After enrollment, patients were randomly divided into a restrictive fluid therapy (RF) group and a GDT group using a computer-generated randomization table. Randomization was assigned in a 1:1 ratio to a trial group. An anesthesiologist administered the study intervention according to the randomization sequence. The anesthesiologist was not blinded to the trials because he/she was responsible for the delivery of the intervention. Outcome assessments and statistical analyses were performed by blinded researchers. The patients, outcome assessor, and surgical team were blinded to the study allocation status of the participants.

### Management of general anesthesia and analgesia

All patients fasted for 8 h and did not receive any sedative or analgesic medications before the induction of anesthesia. After entering the operating room, peripheral venous infusion of the upper limbs and radial arterial lines was established. The multifunctional monitor (model: infinityC700, Germany, Draegerwerk AG&CO. KGaA) was used to continuously monitor the heart rate (HR), arterial blood pressure, mean arterial pressure (MAP), oxygen saturation (SpO_2_), and bispectral index (BIS). For BIS monitoring, a disposable BIS sensor (BIS^TM^ sensor; America, Covidien IIc Co.) was applied to the forehead after the skin was wiped with alcohol swabs. PPV, cardiac index (CI), and stroke volume (SV) were monitored with the continuous noninvasive arterial pressure monitoring system (CNAP) (model: CANP™ Monitor 500, Guangzhou Xinju Science and Trade Co.). For measuring rSO_2_, two sensors for near-infrared spectroscopy (model: egos-600a, Suzhou Aiqin Bio-Medical Electronics Co.) were pasted on the left and right sides of the forehead. The patient’s baseline rSO_2_ data were acquired before anesthetic induction while he/she breathed room air.

One hundred percent oxygen was provided with a mask before intubation. General anesthesia was induced with sufentanil 0.5 μg·kg^−1^, etomidate 0.2 mg·kg^−1^, and rocuronium 0.6 mg·kg^−1^. After successful intubation, all patients received volume-controlled mechanical ventilation with 8 mL·kg^−1^ of tidal volume and an inspiratory-to-expiratory ratio of 1:2, and the respiratory rate was adjusted to maintain the end-tidal pressure of carbon dioxide (P_ET_CO_2_) concentration at 35 to 45 mmHg. Anesthesia was maintained with sevoflurane inhalation (1–2%) in a 2 L 50% oxygen/air mixture, as well as continuous intravenous (IV) propofol (4–8 mg·kg^−1^ h^−1^), remifentanil (0.1–0.3 μg·kg^−1^·min^−1^), and cisatracurium (0.1–0.2 mg·kg^−1^ h^−1^) infusions to keep the BIS values between 40 and 60. The patient’s body temperature was monitored by a nasal thermometer (Shen Zhen, Mecun, Healthcare Co.) and maintained between 36.0°C and 37.0°C. Cisatracurium and sevoflurane were terminated approximately 30 min before the end of the surgery, and 10 μg sufentanil was administered approximately 30 min before skin closure. Propofol and remifentanil were discontinued at the end of the surgery. After surgery, early recovery was managed in the postanesthesia care unit (PACU), and patients were sent back to the ward with a Steward resuscitation score above 4 (Steward 1975). All patients used intravenous patient-controlled analgesia postoperatively for 48 h. The IV patient-controlled analgesia consisted of 2.5 μg·kg^−1^ sufentanil and 2 mg granisetron (total volume of 100 mL, including 0.9% normal saline, basal rate with 0.05 μg·kg^−1^·h^−1^ sufentanil and 0.04 mg·h^−1^ granisetron, bolus with 0.05 μg·kg^−1^ sufentanil and 0.04 mg granisetron, and lockout time 15 min).

### Perioperative fluid and hemodynamic management

RF was designed to a bolus of lactated Ringer’s solution with a dose of 5 mL·kg^-1^ before the induction of anesthesia, followed by a dose of 5 mL·kg^-1^·h^-1^ until the end of surgery. Hypotension (hypotension was defined as MAP ≤ 60 mmHg and/or systolic pressure ≤ 90 mmHg for more than 5 min) was initially treated in the form of an ephedrine bolus of 6 mg with a maximal cumulative dose of 30 mg. If needed, a continuous infusion of norepinephrine (0.05 μg·kg^-1^·min^-1^) was administered (Picard et al. 2016). If hypotension did not respond to vasopressors, fluid boluses of 3 mL·kg^-1^ of lactated Ringer’s solution were given.

**Fig. 1:**
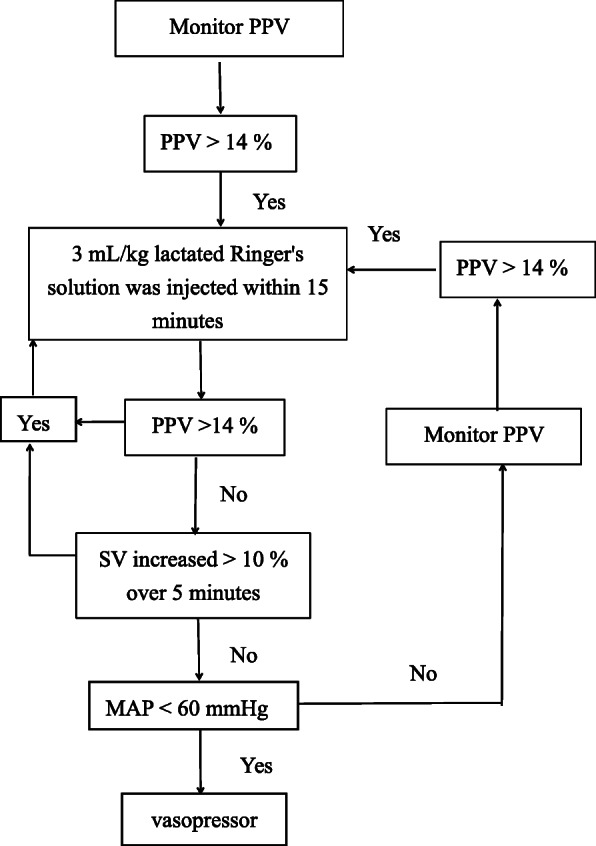
Flow of GDT. PPV pulse pressure variation, SV stroke volume, MAP mean arterial pressure

Patients in group GDT received the fluid management depicted in Fig. 1. The GDT group had a bolus of lactated Ringer’s solution at a dose of 5 mL·kg^-1^ before the induction of anesthesia, followed by a dose of 5 mL·kg^-1^·h^-1^. Under the control of the CNAP system, each time, 3 mL·kg^-1^ of lactated Ringer’s solution within 15 min was injected to maintain PPV ≤ 14%. When the measured PPV was greater than 14% (for 5 min) or when SV increased more than 10%, another 3 mL·kg^-1^ of lactated Ringer’s solution was given until PPV ≤ 14% was realized. The intervention was repeated when PPV remained > 14% following the fluid bolus. When MAP remained < 60 mmHg despite the fluid intervention or MAP was < 60 mmHg with PPV ≤ 14% and SV increased less than 10%, vasopressor therapy was initiated, in the form of an ephedrine bolus of 6 mg with a maximal cumulative dose of 30 mg. If needed, a continuous infusion of norepinephrine (0.05 μg·kg^-1^·min^-1^) was administered (Hasanin et al. 2019).

Blood loss in all patients was replaced with hydroxyethyl starch 130/0.4 sodium chloride injection (HES) at a 1:1 ratio, and blood transfusion was started when clinically indicated and supported by laboratory evidence of hemoglobin less than 70 g·L^-1^. Postoperative fluids for both groups followed an identical regimen: a maintenance rate of 0.5 mL·kg^-1^·h^-1^ lactated Ringer’s solution (minimum 40 mL·h^-1^) for the 6 h after the operation with additional boluses allowed for hypotension, or urine output < 0.5 mL·kg^-1^·h^-1^ for 4 h.

### Outcome measures

The primary endpoint was the incidence of POD at 1–3 days after surgery, which was evaluated once daily at the same time in the afternoon by the same systematic training member who was blinded to the group assignment. POD was assessed with the Confusion Assessment Method-Chinese Revision (CAM-CR) scale, which used the original 11 items of the CAM but developed a 4-point evaluation for each item (Juan et al. 2003; Guo et al. 2017). The items of CAM-CR include acute onset, attention deficit, disorientation, memory deficit, perceptual deficit, excitement, hysteresis, fluctuation of illness, and change in the sleep-wake cycle. Assessment criteria were as follows: a score of < 19 points indicated that the patient did not have delirium, a score of 20–22 points indicated that the patient had suspected delirium, and a score of > 22 points indicated that the patient had delirium.

The total intraoperative fluid infusion volume (including lactated Ringer’s solution and HES), urine volume, estimated blood loss volume (including the blood collected during cell saver and suction canisters), patients received packed red blood cells, cell saver blood administered volume, and dose of vasoactive agents were recorded during the operation. HR, MAP, CI, and rSO_2_ were recorded at 5 min before the induction of anesthesia (T_0_); 5 min after induction of anesthesia (T_1_); start of surgery (T_2_); 30 min, 60 min, 90 min, and 120 min after the start of surgery (T_3_–T_6_); end of surgery (T_7_); and 5 min after extubation (T_8_). Arterial blood lactic acid was collected at T_0_ and T_8_.

Additionally, the duration of anesthesia and surgery, lengths of PACU and hospital stay, VAS pain score, and postoperative complications (including postoperative nausea and vomiting, hypotension, acute renal injury, wound infection, arrhythmia, and pneumonia) were recorded.

### Sample size and statistical analyses

The primary outcome measure was the incidence of POD after spine surgery in the hospital. Based on the past data of our hospital, the incidence of POD was approximately 20.0% in patients who followed RF during spine surgery. According to the research of Zhang et al (Zhang et al. 2018), we assumed that the incidence of POD was approximately 6.67% in patients who followed GDT. A sample size of 97 patients in each group was calculated for a 0.05 difference (two-sided) with a power of 80%. The sample size in the present study was calculated by using PASS 2008 (NCSS, LLC. Kaysville, Utah, USA) software. The final sample size in the present study was determined to be 106 patients per group when considering a 10% dropout rate and higher power.

Statistical analyses were performed using SPSS version 21.0 (SPSS Inc., Chicago, IL, USA). The measurement data were tested for normal distribution with the Kolmogorov-Smirnov test and for homogeneity of variance with the Levene test. Normally distributed qualitative data are presented as the mean (standard deviation), and nonnormal distributions are expressed as the median (interquartile range, IQR). Categorical variables are expressed as numbers (%). Categorical variables including sex, ASA classification, type of surgery, dependence on smoking, alcohol abuse, number of preoperative comorbidity, types of surgery, number of blood transfusion, and the incidence of postoperative complications were analyzed using the *χ*^2^ test or Fisher’s exact tests for smaller events (< 5). Group comparisons of BMI and preoperative hemoglobin were assessed with Student’s *t* test (normally distributed qualitative data). Group comparisons of age, BMI, preoperative waiting time, preoperative HCT, length of PACU stay and hospital stay, postoperative VAS pain score, number of vertebrae involved in the surgery, anesthesia duration, surgery duration, volume of fluid infusion, amount of bleeding, volume of urine output, dosage of vasopressor, hypotensive events, and lactate acid were performed using the Mann–Whitney *U* test (non-normally distributed qualitative data). Repeated measurement analysis of variance was used to analyze the intraoperative data of rSO_2_, CI, MAP, HR, and BIS.

## Results

**Fig. 2:**
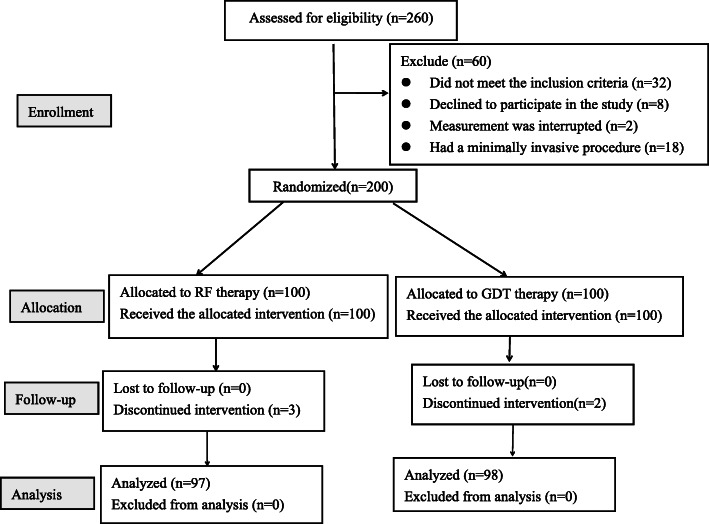
Flow chart of the patients studied. RF restrictive fluid therapy, GDT goal-directed fluid therapy

A total of 260 patients with spinal surgery were screened in this study from May 2020 to November 2020. 228 patients met the inclusion criteria, and 220 patients were given consents and subsequently randomly assigned to the RF group or the GDT group. During the study period, 25 patients were excluded (18 patients who had a minimally invasive lumbar procedure, 3 patients who changed their fluid therapy due to severe hypotension, 2 patients who experienced severe complications during surgery, and 2 patients who did not complete the study). Ninety-seven patients in the RF group and 98 patients in the GDT group were included in the final analysis (Fig. 2).

### Patient characteristics and operative data

No differences in age, gender, BMI, ASA, nicotine dependence, alcohol abuse, preoperative comorbidity, education years, preoperative hemoglobin, types of surgery, preoperative waiting time, number of patients using nonsteroidal anti-inflammatory drugs (NSAIDs), or number of vertebrae involved in the surgery were observed between the two groups (Table 1).

**Table 1:** Demographic characteristics of the study patients

Characteristic	Group RF (***n*** = 97)	Group GDT (***n*** = 98)	***P*** value
**Age, med (IQR)**	60 (55–68)	58 (56–66)	0.929
**Male,** ***n*** **(%)**	52 (53.6)	52 (53.1)	0.939
**BMI (kg·m**^**−2**^**), mean (SD)**	23.54 (3.40)	23.80 (2.75)	0.547
**ASA**			
I, *n* (%)	28 (28.9)	30 (30.6)	0.790
II, *n* (%)	69 (71.1)	68 (69.4)	0.790
**Education,** ***n*** **(%)**			
Illiterate, *n* (%)	30 (30.9)	30 (30.6)	0.718
1–6 years of education years, *n* (%)	24 (24.7)	29 (29.5)	0.718
≥7 years of education years, *n* (%)	43 (44.3)	39 (39.7)	0.718
**Baseline MMSE, med (IQR)**	29 (25–30)	27 (25–30)	0.348
**Dependence on smoking,** ***n*** **(%)**	19 (19.5)	18 (18.3)	0.828
**Alcohol abuse,** ***n*** **(%)**	7 (7.2)	11 (11.2)	0.334
**Comorbidity,** ***n*** **(%)**			
Coronary heart disease, *n* (%)	7 (7.2)	6 (6.1)	0.759
Hypertension, *n* (%)	18 (19.6)	23 (23.4)	0.400
Diabetes mellitus, *n* (%)	7 (7.2)	7 (7.1)	0.984
Hydrothorax, *n* (%)	8 (8.2)	7 (7.1)	0.772
**Type of surgery**			
Lumbar spine stenosis surgery, *n* (%)	69 (71.1)	71 (72.4)	0.838
Thoracic spinal stenosis surgery, *n* (%)	28 (28.9)	27 (27.6)	0.838
**Number of vertebral levels**^***c***^***,*** **med (IQR)**	2 (2–3)	2 (2–3)	0.797
**Preoperative waiting time (d), med (IQR)**	6 (5–8)	6 (5–8)	0.418
**Preoperative hemoglobin (mg/l), mean (SD)**	130.3 (12.8)	131.1 (17.1)	0.710
**Preoperative hematocrit (%), med (IQR)**	40 (37–42)	40 (37–43)	0.567
**Number of patients using NSAIDs,** ***n*** **(%)**	11 (11.3)	12 (14.3)	0.538

*Med* median, *IQR* interquartile range, *SD* standard deviation, *ASA* American Society of Anesthesiologists, *BMI* body mass index, *med* median, ^*c*^Levels were defined as the number of vertebrae involved in the surgery, *NASIDs* nonsteroidal anti-inflammatory drugs

The intraoperative data of surgery and lactated Ringer’s solution volume within 6 h after the operation are presented in Table 2. No differences in anesthesia duration, surgery duration, the length of PACU stay, patients received packed red blood cells, cell saver blood administered volume, and the lactated Ringer’s solution volume within 6 h after operation were found between the two groups. Compared with group RF, group GDT had a higher volume of intraoperative lactated Ringer’s solution [1500 (1128 to 1775) mL versus 1000 (765 to 1300) mL, *P* < 0.001) and total intraoperative fluid infusion [1700 (1396 to 2200) mL versus 1280 (961 to 1611) mL, *P* < 0.001], but there was no difference in HES infused volume and amount of bleeding between the two groups. Patients in group RF were more likely than those in group GDT to have lower urine output [300 (200 to 530) mL versus 398 (288 to 600) mL, *P* = 0.012], receive more vasopressor support (*P* = 0.015), experience more hypotensive events (*P* < 0.001), and prolong the length of hospital stay [17.0 (14 to 20) days versus 14.5 (13 to 17.0) days, *P* = 0.001] (Table 2).

**Table 2:** Surgical data with outcomes

	Group RF (***n*** = 97)	Group GDT (***n*** = 98)	***P*** value
**Anesthesia duration (min), med (IQR)**	195 (172–232)	205 (163–240)	0.918
**Surgery duration (min), med (IQR)**	167 (141–203)	176 (137–218)	0.963
**Total intraoperative fluid volume**	1280 (961–1611)	1700 (1396–2200)	<0.001
Intraoperative lactated Ringer's solution volume (mL), med (IQR)	1000 (765–1300)	1500 (1128–1775)	<0.001
Intraoperative HES (mL), med (IQR)	300 (200–440)	300 (250–500)	0.312
**Intraoperative hypotensive event, med (IQR)**	1 (0–2)	0 (0–1)	<0.001
**Intraoperative ephedrine dose (mg), med (IQR)**	6 (0–12)	0 (0–6)	0.015
**Intraoperative urine output (mL), med (IQR)**	300 (200–530)	398 (288–600)	0.012
**Intraoperative blood transfusion**			
Patients receiving packed RBCs, n (%)	8 (8.2)	6 (6.1)	0.565
Cell saver blood (mL), med (IQR)	0 (0–125)	0 (0–134)	0.661
**Intraoperative estimated blood loss (mL), med (IQR)**	270 (200–320)	268 (200–351)	0.520
**Length of PACU stay (min), med (IQR)**	83 (65–113)	78 (64–98)	0.407
**Length of hospital stay (days), med (IQR)**	17.0 (14.0–20.0)	14.5 (13.0–17.0)	<0.001
**Lactated Ringer’s solution volume within 6 h after operation(mL), med (IQR)**	372 (339–435)	390 (342–450)	0.275

Anesthesia duration refers to the time from the beginning of anesthesia to the end of anesthesia. Surgery duration refers to the time from the beginning of surgery to the end of surgery

*PACU* postanesthesia care unit, *RBC* red blood cell

### Primary endpoint

The overall incidence of POD was approximately 8.2% in the first 3 days after spine surgery in middle- and old-aged patients. Postoperative delirium occurred in 12 (12.4%) of 97 patients given restrictive fluid therapy and in 4 (4.1%) of 98 patients given GDT (odds ratio [OR] 0.301, 95% CI 0.094–0.970; *P* = 0.035) during 3-day follow-up. Patients in group GDT showed a lower prevalence of POD on postoperative days 1–2 than patients in group RF (day 1: 10.3% versus 3.1%, *P* = 0.042; day 2: 9.3% versus 2.0%, *P* = 0.029). There was no significant difference in the prevalence of POD on the third day between the group RF and the group GDT (Table 3).

**Table 3:** Daily prevalence of postoperative delirium

	Group RF (***n*** = 97)	Group GDT (***n*** = 98)	***P*** value
**POD**			
1 day postoperative, *n* (%)	10 (10.3)	3 (3.1)	0.042
2 days postoperative, *n* (%)	9 (9.3)	2 (2.0)	0.029
3 days postoperative, *n* (%)	3 (3.1)	1 (1.0)	0.369

*POD* postoperative delirium

### Secondary endpoints and postoperative characteristics

**Fig. 3:**
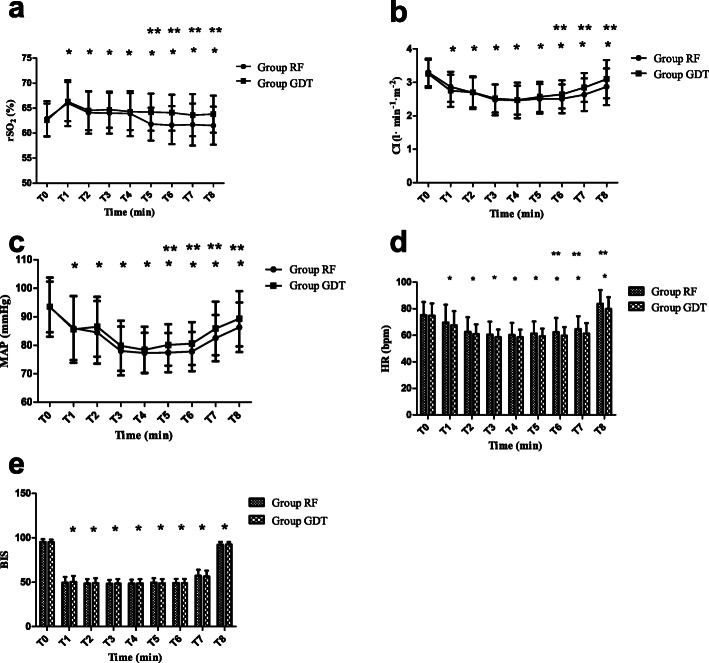
Continuous variables are presented as mean (standard deviation). Compared with T_0_, rSO_2_ in group RF was increased at T_2_–T_4_ but decreased at T_5_–T_8_, while compared with T_0_, rSO_2_ in group GDT was increased at T_1_–T_8._ Compared with T_0_, MAP, CI, and BIS in both groups were decreased at T_1_–T_8_. Compared with T_0_, HR in both groups was decreased at T_1_–T_7_ but increased at T_8_ (^*^P < 0.05). Compared with group RF, intraoperative rSO_2_ and MAP values were increased at T_5_–T_8_ in group GDT (^**^P < 0.05) (**a**, **c**); compared with group RF, intraoperative CI and HR values were increased at T_6_–T_8_ in group GDT (^**^P < 0.05) (**b**, **d**). T_0_: 5 min before the induction of anesthesia; T_1_: 5 min after induction of anesthesia; T_2_: start of surgery; T_3_: 30 min after the start of surgery; T_4_: 60 min after the start of surgery; T_5_: 90 min after the start of surgery; T_6_: 120 min after the start of surgery; T_7_: end of surgery; T_8_: 5 min after extubation. MAP mean arterial pressure, *rSO*_*2*_ regional cerebral oxygen saturation, *HR* heart rate, CI cardiac index, *BIS* bispectral index

Compared with T_0_, rSO_2_ in group RF was increased at T_2_–T_4_ but decreased at T_5_–T_8_, while compared with T_0_, rSO_2_ in group GDT was increased at T_1_–T_8_ (*P* < 0.05). Compared with T_0_, MAP, CI, and BIS in both groups were decreased at T_1_–T_8_ (*P* < 0.05). Compared with T_0_, HR in both groups was decreased at T_1_–T_7_ but increased at T_8_ (*P* < 0.05). Compared with group RF, MAP and rSO_2_ were increased at T_5_–T_8_ in group GDT. Compared with group RF, CI and HR were increased at T_6_–T_8_ in group GDT (*P* < 0.05). There was no significance in BIS values between group RF and group GDT (*P >* 0.05) (Fig. 3).

The intraoperative change in lactate (postoperative compared to preoperative) in the RF group ranged from −0.5 to 1.5 mmol/L with a median of 0.2 (interquartile range 0.0 to 0.3), and in the GDT group, this change ranged from −1.2 to 0.9 mmol/L with a median of −0.1 (interquartile range −0.3 to 0.0). Although there was a statistical significance in postoperative lactic acid level between group RF and group GDT (*P* < 0.05, Table 4), it had a lower power for clinical significance.

**Table 4:** Preoperative and postoperative blood tests and postoperative complications

	Group RF (***n*** = 97)	Group GDT (***n*** = 98)	***P*** value
**VAS pain score, med (IQR)**			
24 h postoperatively, med (IQR)	3 (2–4)	3 (2–4)	0.753
48 h postoperatively, med (IQR)	2 (2–3)	2 (2–3)	0.854
72 h postoperatively, med (IQR)	2 (1–2)	2 (1–3)	0.855
**Preoperative creatinine (μmol/l), med (IQR)**	57 (49–68)	59 (48–73)	0.672
**Postoperative creatinine (μmol/l), med (IQR)**	57 (49–78)	56 (44–66)	0.074
**Preoperative lactic acid, med (IQR)**	1.2 (0.7–1.6)	1.2 (0.9–1.7)	0.393
**Postoperative lactic acid, med (IQR)**	1.3 (1.0–1.8)	1.2 (0.8–1.4)	0.001
**Change of lactic acid, med (IQR)**	0.2 (0.0–0.3)	-0.1 (-0.3–0.0)	0.000
**Complications,** ***n*** **(%)**			
PONV, *n* (%)	13 (13.4)	16 (16.3)	0.566
Hypotension, *n* (%)	5 (5.2)	1 (1.0)	0.118
Acute renal injury, *n* (%)	4 (4.1)	1 (1.0)	0.212
Wound infection, *n* (%)	2 (2.0)	0 (0.0)	0.246
Arrhythmia, *n* (%)	3 (3.1)	1 (1.0)	0.369
Pneumonia, *n* (%)	1 (1.0)	1 (1.0)	1.000

*VAS* visual analog scale, *PONV* postoperative nausea and vomiting, *Change of lactic acid* postoperative lactic acid–preoperative lactic acid

Postoperative VAS pain scores and postoperative complications, including postoperative nausea and vomiting, hypotension, acute renal injury, wound infection, arrhythmia, and pneumonia (Table 4), were compared between the RF group and the GDT group. Compared with group RF, the incidence of hypotension showed an increasing trend in the restrictive group (5 versus 1, *P* = 0.118), though this trend was not clinically significant (Table 4).

## Discussion

This prospective randomized study found that the POD incidence for middle- and old-aged patients in the first 3 days after spine surgery was 8.2%. GDT compared with RF was found to be associated with a reduced of incidence of POD in the first 3 days after surgery and a reduced prevalence of POD on postoperative days 1–2.

In our study, the incidence of POD in the RF group was 12.4%, relatively lower than the historical incidence of POD at our institution. The reasons that led to the low delirium incidence in the current patient population may include the following. Firstly, none of the patients received any sedative medications before the induction of anesthesia, especially benzodiazepines often used as preoperative routine medication in our institution, which may increase the incidence of POD (Poeran et al. 2020). Secondly, our study excluded patients with visual, auditory, or language communication disorders, and those with any significant comorbid disease. These patients were at a great increased risk of delirium (Inouye et al. 2014; Jung et al. 2018; Aldecoa et al. 2017).

Spinal surgery often needs to be performed in the prone position, and dramatic changes in position will lead to hemodynamic instability (Bacchin et al. 2016). Recently, studies have shown that GDT can provide stable hemodynamics, effectively optimize the relationship between tissue perfusion and volume loads, and improve postoperative outcomes (Cannesson 2010; Tapia et al. 2021; Bacchin et al. 2016; Zhang et al. 2018). To our knowledge, this study is one of the few studies to directly compare the effects of intraoperative RF with PPV-directed fluid therapy on the incidence of POD in patients undergoing spinal surgery. Our results suggest that PPV-directed fluid therapy results in a reduced incidence of POD and a shorter length of hospital stay than RF. However, the incidence of other postoperative complications was similar between the two groups.

The accuracy and early recognition of the intravascular volume status are essential to prevent both hypoperfusion due to volume depletion and fluid overload due to an unnecessary infusion (Zhang et al. 2012), which may promote the occurrence of POD. Therefore, appropriate hemodynamic monitoring is necessary for intraoperative fluid management. A simple, affordable, and reliable method to achieve this goal would be appropriate for the routine intraoperative application. The PPV based on blood pressure waveforms detected with the CNAP system has been shown to be reliable (Jeleazcov et al. 2010; Biais et al. 2017). This type of PPV monitoring is not associated with additional costs or complications other than arterial catheterization. Benes et al. (Benes et al. 2015) demonstrated that intraoperative PPV-guided fluid therapy during total knee and hip replacement surgery improved postoperative outcomes. PPV has also been validated in the prone position: a study from Yang et al. (Yang et al. 2013) demonstrated that PPV can reliably predict fluid responsiveness in the prone position, albeit with a lower threshold than in supine position (15% vs 14%). According to Yang’s (Yang et al. 2013) study, we used a PPV > 14% as a trigger for intraoperative administration of a fluid bolus to target volume optimization in this study.

Many studies have investigated the effects of the amount of intraoperative fluid administration on perioperative outcomes. Studies have demonstrated that fluid overload increases the incidence of perioperative complications in spinal surgery, such as POD (Brown et al. 2016) and pulmonary complications (Siemionow et al. 2012a, b), whereas RF yields better outcomes, including fewer postoperative complications and a shorter length of hospital stay (Brandstrup et al. 2003; Hart et al. 2013; Shin et al. 2018). A study of cervical decompression and fusion across the cervicothoracic junction reported that intraoperative restriction of IV fluid while maintaining adequate blood pressure reduces airway complications in this patient population (Hart et al. 2013). Additionally, RF, including avoidance of synthetic colloids to reduce dilution and colloid-induced coagulopathy and increased bleeding, is a crucial element of patient blood-management strategies, while patient blood management has been reported to reduce the need for red blood cell (RBC) transfusion in idiopathic scoliosis posterior spinal surgery (Ohrt-Nissen et al. 2017). However, intraoperative fluid restriction is associated with frequent episodes of intraoperative hypotension, which is a major determinant of postoperative organ dysfunction (Zhang et al. 2012), and hypotension may be a risk factor of POD development (Patti et al. 2011; Maheshwari et al. 2020). In addition, several studies have examined the effects of intraoperative GDT on perioperative outcomes. Researchers have demonstrated that GDT reduces the incidence of POD and the duration of hypotensive episodes in major spine surgery (Picard et al. 2016; Zhang et al. 2018). A meta-analysis including six trials with a total of 562 participants found that based on very low-certainty evidence, RF may increase the risk of all-cause mortality compared to GDT, but the evidence is very uncertain (Wrzosek et al. 2019). Based on very low-certainty evidence, whether GDT is superior to a restrictive fluid strategy in major spinal surgery remains uncertain. In our study, patients who were treated using GDT received greater amounts of fluid than patients who were treated with RF. Additionally, MAP at T_5_–T_8_ and CI at T_6_–T_8_ in the GDT group were higher than those in the RF group, and intraoperative hypotensive events were lower in the GDT group than in the RF group, indicating that patients undergoing major spinal surgery may benefit from GDT (compared with RF), with a reduction in intraoperative hypotensive events and the incidence of POD.

Some studies have suggested that intraoperative hemodynamic fluctuation may result in transient cerebral hypoperfusion (Hirsch et al. 2015; van Waes et al. 2016), which may promote the incidence of POD. Previous studies found that intraoperative hypotension was significantly associated with developing POD (Patti et al. 2011; Maheshwari et al. 2020). Significant intraoperative hypotension often necessitates the use of vasoactive medications and several studies have found that POD was associated with higher intraoperative vasopressor usage (Rudiger et al. 2016; Neerland et al. 2017). In our study, patients in group GDT were more likely than those in group RF to maintain more stable perioperative hemodynamics, experience lower intraoperative hypotensive events, receive more vasoactive drug, and have a lower incidence of POD, indicating that GDT may maintain hemodynamic stability and avoid intraoperative hypotension to prevent cerebral hypoperfusion, thereby reducing the incidence of POD.

Recently, a meta-analysis suggested that early GDT improves tissue perfusion and oxygenation in high-risk patients undergoing major abdominal and orthopedic surgery (Giglio et al. 2019). Accumulating evidence suggests that rSO_2_ reflects the overall cerebral tissue oxygen supply/demand (Fischer and Silvay 2010; Rescoe et al. 2017), and higher rSO_2_ desaturation is significantly associated with POD in certain types of surgery (Green and Kunst 2017; Wang et al. 2019). In our study, we used rSO_2_ to reflect the effect of intraoperative fluid therapy on POD. In this study, there was no statistically significant difference in preoperative hemoglobin content between the two groups, and the inhaled oxygen concentration was controlled at 50% during surgery. Perioperative P_ET_CO_2_ was controlled at 35–45 mmHg, and the intraoperative body temperature was maintained at a constant level to eliminate interference by other factors in the rSO_2_ value. In our study, rSO_2_ in the GDT group was higher than that in the RF group after 90 min of surgery, and the incidence of POD was lower than that in the RF group, indicating that compared with RF, GDT can improve rSO_2_ in spinal surgery, which may be helpful to reduce the incidence of POD.

There are many factors affecting POD, such as age, BMI, ASA score, and postoperative pain (Radinovic et al. 2019). In this study, there were no statistically significant differences in age, ASA classification, BMI, or postoperative pain, which ensures the comparability of the two groups. A previous study also suggested that hydroxyethyl starch administration was related to early POD (Jung et al. 2018). In this study, there were no statistically significant differences in hydroxyethyl starch administration between the two groups, which endure the comparability of the two fluid therapies. The meta-analysis reported that BIS at different depths may influence POD (Bocskai et al. 2020). In this study, there were no statistically significant differences in BIS values.

Nonetheless, there were a few limitations in this study. Firstly, we excluded seven patients (3 patients changed their fluid therapy due to severe hypotension in the RF group, 2 patients who experienced severe complications during surgery in the GDT group, and 1 patient in each group who did not complete the study) in analysis. Secondly, GDT was only performed during the intraoperative period, the application time was short, and there was no long-term follow-up. Thirdly, only RF and GDT were compared, and they were not compared with liberal fluid therapy. Fourthly, there was a lack of blood indicators to monitor POD, such as S100β, IL-6, and IGF-I. Furthermore, considering that this study was a small sample size, single-center, and patients with strict and extensive inclusion and exclusion criteria, the extrapolation of our study to other populations was limited. Thus, further prospective studies with larger populations and longer follow-up times are required to compare the effects of RF, traditional fluid therapy, and GDT on delirium.

## Conclusion

In summary, GDT can reduce the incidence of POD and shorten the length of hospital stay in middle- and old-aged patients undergoing spine surgery, and it may be related to maintain the stability of perioperative hemodynamics and increased cerebral oxygen supply.

## Data Availability

The datasets used and/or analyzed during the current study are available from the corresponding author on reasonable request.

## Abbreviations

POD: Postoperative delirium
GDT: Goal-directed fluid therapy
RF: Restrictive fluid therapy
CNAP: Continuous noninvasive arterial pressure
PPV: Pulse pressure variation
rSO_2_: Regional cerebral oxygen saturation
MAP: Mean arterial pressure
HR: Heart rate
CI: Cardiac index
VAS: Visual analog scale
SV: Stroke volume
P_ET_CO_2_: End-tidal pressure of carbon dioxide
IV: Intravenous
PACU: Postanesthesia care unit
HES: Hydroxyethyl starch 130/0.4 sodium chloride injection
CAM-CR: Confusion Assessment Method-Chinese Reversion Scale
IQR: Interquartile range
BMI: Body mass index
ASA: American Society of Anesthesiologists
PONV: Postoperative nausea and vomiting
RBC: Red blood cell
